# Comparative Diminution of Patulin Content in Apple Juice With Food-Grade Additives Sodium Bicarbonate, Vinegar, Mixture of Sodium Bicarbonate and Vinegar, Citric Acid, Baking Powder, and Ultraviolet Irradiation

**DOI:** 10.3389/fphar.2018.00822

**Published:** 2018-08-13

**Authors:** Minkyeong Kim, Shruti Shukla, Youngsook Oh, Soo Hyun Chung, Myunghee Kim

**Affiliations:** ^1^Department of Food Science and Technology, Yeungnam University, Gyeongsan, South Korea; ^2^Department of Energy and Materials Engineering, Dongguk University, Seoul, South Korea; ^3^Department of Integrated Biomedical and Life Science, Korea University, Seoul, South Korea

**Keywords:** apple juice, patulin, reduction, sodium bicarbonate, method development

## Abstract

This study aimed to determine an optimal method for patulin (PAT) reduction for application in apple juice production. PAT levels in spiked apple juice (100 μg/L) were measured after treatment with citric acid, sodium bicarbonate, vinegar, mixture of sodium bicarbonate and vinegar, baking powder, and ultraviolet (UV) irradiation. Treatments with sodium bicarbonate and UV irradiation were most effective in reducing PAT; however, UV irradiation reduced the yellowness (*b*^∗^) of apple juice. However, sodium bicarbonate treatment affected quality attributes including soluble solids, pH, and color of apple juice. The color and odor of apple juice treated with sodium bicarbonate could be recovered *via* addition of citric acid. The present results suggest that sodium bicarbonate could be considered an additive in apple juice for PAT reduction.

## Introduction

Mycotoxins are toxic secondary fungal metabolites. Patulin (PAT) is a mycotoxin produced by various fungal species of genera *Penicillium*, *Aspergillus*, and *Byssochlamys* (Paster et al., 1995; Shephard and Leggott, 2000). PAT is present in various rotten fruits including apple, pear, strawberry, blueberry, peach, and apricot, and in few moldy vegetables (Gökmen and Acar, 1998; Zouaoui et al., 2015). PAT has especially raised considerable concern among consumers owing to contamination of apple juice.

**FIGURE 1:**
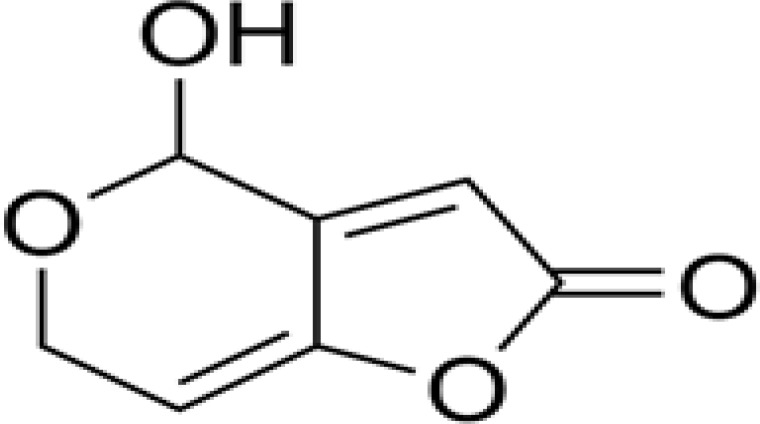
Chemical structure of patulin.

Patulin is a white crystalline substance with a molecular formula of C_7_H_6_O_4_ (**Figure 1**) and a molecular weight of 154.13; it is soluble in water, methanol, ethanol, acetone, ethyl acetate, and amyl acetate, but insoluble in diethyl ether or benzene (Ministry of Food and Drug Safety, 2010). PAT is stable at a pH < 6 but hydrolyzed in alkaline solutions (Ministry of Food and Drug Safety, 2010). PAT may cause neurotoxicity, DNA damage, and adverse effects on the gastrointestinal tract in rodents (Ministry of Food and Drug Safety, 2010), thereby giving rise to concerns regarding similar potential effects in humans (Marin et al., 2011). The International Agency for Research on Cancer (1993) classified PAT into group 3, not classifiable as carcinogenic to humans. Owing to its toxicity, its maximum tolerable daily intake has been set as 0.4 μg/kg body weight by the Joint FAO/WHO Expert Committee on Food Additives (1995).

Nowadays, consumption of organic apple has gained considerable popularity owing to the perception of health and wellness benefits associated with organically grown food (Pique et al., 2013). Organic apple production entails strict limits on pesticide use (Pique et al., 2013), thereby increasing susceptibility to damage by fungal pathogens, which produce PAT. This may lead to PAT contamination in juice produced from the organic apples. Moreover, depectinization, clarification, and filtration steps in apple juice processing eliminate less than half of the initial amount of PAT (Acar et al., 1998). Additionally, insufficient application of Good Agricultural Practices and Good Manufacturing Practices during pre- and post-harvest of rotten apples results in high concentrations of PAT in apple and apple products (Zhu, 2014). Furthermore, the standard pasteurization procedure for apple juice (72°C for 6 s) cannot eliminate PAT completely (Wheeler et al., 1987). Hence, the Codex Alimentarius, European Commission established a maximum permissible concentration of 50 μg/kg PAT in apple juice and apple products (FAO/WHO Codex Alimentarius Commission, 2003; European Commission, 2006).

Ultraviolet (UV) irradiation has been used previously for PAT reduction by 90% in apple juice (Zhu et al., 2014). However, UV irradiation affects reduction of ascorbic acid of apple juice (Zhu et al., 2014). In addition, activated carbon (3 g/L) was previously reported to reduce PAT in apple juice by 97% after 30 min of reaction (Fathi-Achachlouei et al., 2007); however, the major drawback of this study was the use of a double filtration process and undesirable changes in color values, thereby deterring the actual processing of apple juice.

To overcome these limitations of previous studies on PAT reduction, we assessed various types of Generally Regarded as Safe (GRAS) grade material additives (citric acid, sodium bicarbonate, vinegar, mixture of sodium bicarbonate and vinegar, and baking powder; United States of Food and Drug Administration, 2000, 2016a,b), which are expected to reduce PAT in apple juice, for their practical application in the apple juice industry owing to their reportedly efficient antimicrobial activity.

## Materials and Methods

### Materials and Chemicals

Clear apple juice, vinegar, and baking powder were obtained from local super market in Korea. Citric acid was purchased from Wako Pure Chemical (Osaka, Japan). PAT standard solution was purchased from Sigma-Aldrich (St. Louis, MO, United States). High-performance liquid chromatography (HPLC)-grade ethyl acetate and acetic acid were purchased from Fisher Chemicals (Pittsburgh, PA, United States) and J. T. Baker (Center Valley, PA, United States), respectively. Acetonitrile of ACS grade was acquired from Merck (Billerica, MA, United States). Sodium bicarbonate and sodium carbonate were acquired from Duksan Pure Chemicals (Ansan, Gyeonggi-do, South Korea). Sodium sulfate was provided by OCI Company (Seoul, South Korea).

### Treatment of Citric Acid, Sodium Bicarbonate, Vinegar, a Mixture of Sodium Bicarbonate and Vinegar, and Baking Powder in Spiked Apple Juice

Patulin standard solution (100 μg/mL) was diluted 10-folds with acetonitrile. Twenty milliliters of apple juice was spiked with 200 μL of diluted PAT solution. PAT concentration in apple juice was adjusted to 100 ng/mL. Different concentrations (1, 5, and 10%) of citric acid, sodium bicarbonate, vinegar, a mixture of sodium bicarbonate and vinegar, and baking powder were added to PAT spiked apple juice samples and further reacted in a rotary incubator (150 rpm) at 25°C for 12 h. Thereafter, the samples were filtered through a 2.5 μm filter paper and the filtrate was used for PAT extraction. All experiments were performed in triplicate.

### UV Irradiation on Apple Juice

Apple juice (20 mL) was transferred to a Petri-dish (diameter, 90 mm; depth, 16 mm) and spiked with 200 μL of 10 μg/mL PAT solution. These samples were UV irradiated at a range of 200–280 nm. The irradiation distance from the lamp to the surface of the samples was 11 cm, in accordance with Ibarz et al. (2014). The samples were UV irradiated for 5, 10, 30, 60, 120, 240, 480, and 720 min under this condition. Thereafter, a 5 mL sample was harvested and used for PAT extraction. All experiments were temporally coordinated and performed in triplicate.

### PAT Extraction

Apple juice (5 mL) was treated with citric acid, sodium bicarbonate, vinegar, mixture of sodium bicarbonate and vinegar, baking powder, and UV irradiation, and was then transferred into a centrifuge bottle; 10 mL of ethyl acetate was then added. This mixture was centrifuged at 4,500 rpm for 5 min at 25°C (Zouaoui et al., 2015). Thereafter, the upper layer was transferred into a 100 mL separating funnel (I). Ethyl acetate (10 mL) was transferred into the centrifuge bottle and centrifuged at 4,500 rpm, for 5 min at 25°C. The upper layer was transferred to the separating funnel (I) again, and the process was repeated. Two milliliters of 0.5% sodium carbonate was pipetted into the separating funnel (I) and subjected to vigorous agitation, and then the lower layer was transferred to another separating funnel (II). Ethyl acetate (3 mL) was transferred into the separating funnel (II) and subjected to agitation. The lower layer of the separating funnel (II) was drained, and the supernatant was combined with the supernatant from the separating funnel (I). Anhydrous sodium sulfate (0.5 g) was added to the separating funnel (II) and subjected to agitation. The mixture was filtered through a filter paper (2.5 μm pore size). The separating funnel (II) was washed with 5 mL of ethyl acetate and filtered through a filter paper (2.5 μm pore size). All filtrate was evaporated with nitrogen gas at 60°C. Thereafter, the residue was dissolved in acetic acid solution of pH 4, which was adjusted using acetic acid. This reconstituted extract was filtered through a syringe filter (0.22 μm pore size) (AOAC, 2000). Extraction was performed at least 10 times to achieve optimal and reproducible PAT recovery.

### HPLC Conditions

Patulin thus extracted was quantified *via* HPLC, using an Dionex Ultimate 3000 UHPLC system (Thermo Scientific, Sunnyvale, CA, United States), equipped with a UV detector (λ = 276 nm) and auto-injector. Separation was achieved on a Sunfire C_18_ column (4.6 mm × 250 mm × 5 μm; Waters; Milford, MA, United States). As a mobile phase, a mixture of water and acetonitrile (95:5, v/v) was pumped at the flow rate of 0.5 mL/min. Standard solutions of PAT (0, 50, 100, 200, and 500 ppb) and sample solutions of PAT extract were filtered through a 0.2 μm pore size of membrane filter (Whatman, GE Healthcare; Kent, England) prior to HPLC analysis, and 20 μL of each sample was injected for a 20-min run-time (AOAC, 2000). Chromatograms were analyzed *via* comparisons with the standard curve of PAT.

### Validation of Analytical Methods

The analytical HPLC procedure was validated *via* calibration and evaluation of the range of linearity, limit of detection (LOD), limit of quantification (LOQ), and recovery rate. Calibration was carried out with PAT standard solutions. The linear response of PAT was determined in the concentration range of 50–700 ppb, which led to the correlation factor *R*^2^ > 0.99. LOD and LOQ were calculated using the equations LOD = *X*_0_ + 3SD and LOQ = *X*_0_ + 5SD, respectively, where *X*_0_ was the average response of the blank samples, and SD referred to the standard deviation for *n* = 6.

### Measurement of Soluble Solids, pH, Turbidity, and Color Change in Apple Juice

Soluble solids in apple juice treated with citric acid, sodium bicarbonate, vinegar, mixture of sodium bicarbonate and vinegar, and baking powder were quantified using a digital refractometer (Hanna Instrument Company; Woonsocket, RI, United States). To determine pH, Orion Star A211 pH meter (Thermo Scientific; Waltham, MA, United States) was used. Turbidity was analyzed at 600 nm with Infinite 200 Microplate Reader (Tecan; Grödig, Austria). Color change (*L*^∗^, *a*^∗^, and *b*^∗^) was measured with CR-400/410 series from Konica Minolta (Tokyo, Japan). *L*^∗^ value indicated lightness representing dark to light, whereas *a*^∗^ and *b*^∗^ values represented the degree of red–green and yellow–blue color, respectively.

### Safety Considerations

All analyses with PAT and its waste treatments were handled in accordance with the institutional safety procedures laws at Yeungnam University, South Korea.

### Statistical Analysis

Statistical analysis was performed using the IBM SPSS software (SPSS Inc.; Chicago, IL, United States). Duncan’s multiple test was conducted, and *p* < 0.05 was considered statistically significant.

## Results

### Effect of Citric Acid, Sodium Bicarbonate, Vinegar, a Mixture of Sodium Bicarbonate and Vinegar, and Baking Powder for Reducing PAT Content in Apple Juice

**FIGURE 2:**
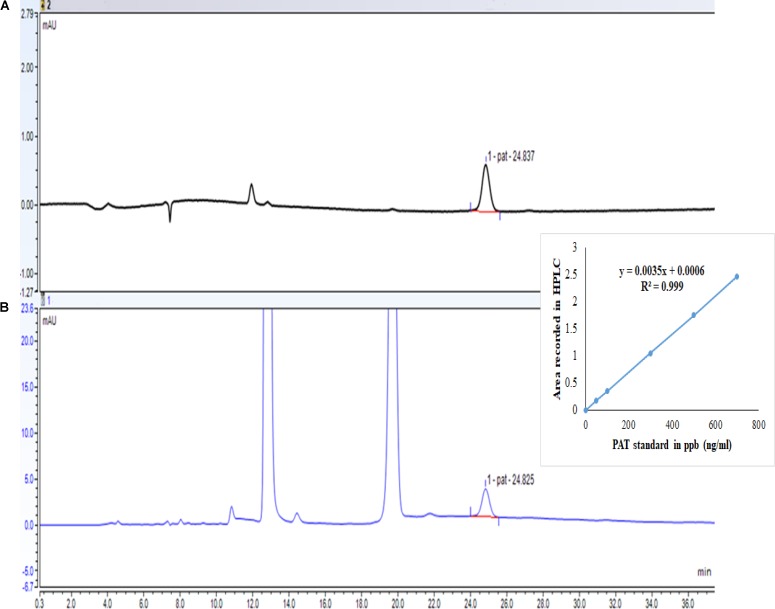
Chromatograms of high-performance liquid chromatography: (A) patulin in acetonitrile as a buffer system (100 μg/L); (B) patulin in apple juice (100 μg/L). The figure inset shows the calibration curve.

**FIGURE 3:**
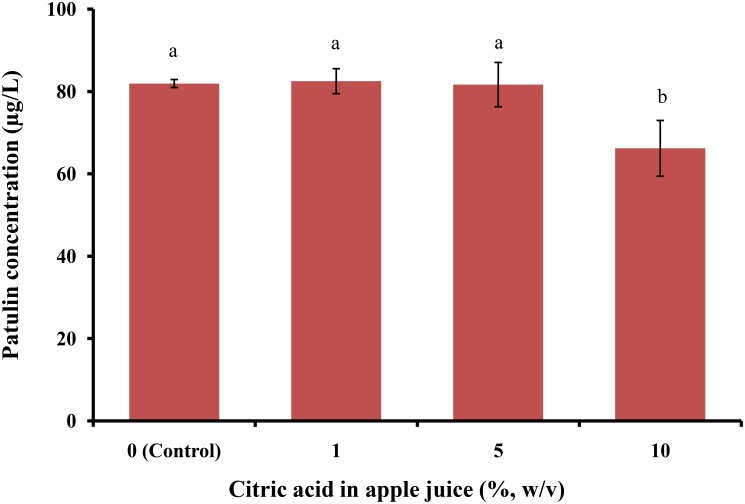
Patulin content in apple juice after treatment with citric acid. Bars represent the standard deviation of the mean of three independent experiments. Lowercase letters indicate significant differences at *p* < 0.05.

Chromatograms for HPLC for standard PAT detected in acetonitrile buffer system (100 μg/L) and for PAT in spiked apple juice (100 μg/L) are shown in **Figure 2**. The retention time for PAT was approximately 24.8 min. Comparative effects of citric acid, sodium bicarbonate, vinegar, a mixture of sodium bicarbonate and vinegar, and baking powder at different concentrations (1, 5, and 10%) were determined in apple juice samples spiked with 100 μg/L PAT for 12 h of incubation at 25°C and 150 rpm for significant reduction of PAT content without affecting the quality parameters of apple juice samples. Consequently, spiked apple juice without addition of citric acid served as a control and was determined 81.92 ± 0.97 μg/L (**Figure 3**). Apple juice treated with 1, 5, and 10% citric acid had reduced concentration of PAT to 82.49 ± 3.03, 81.66 ± 5.38, and 66.18 ± 6.77 μg/L, respectively, confirming that 10% citric acid treatment yielded a significant difference from the control (*p* < 0.05).

**FIGURE 4:**
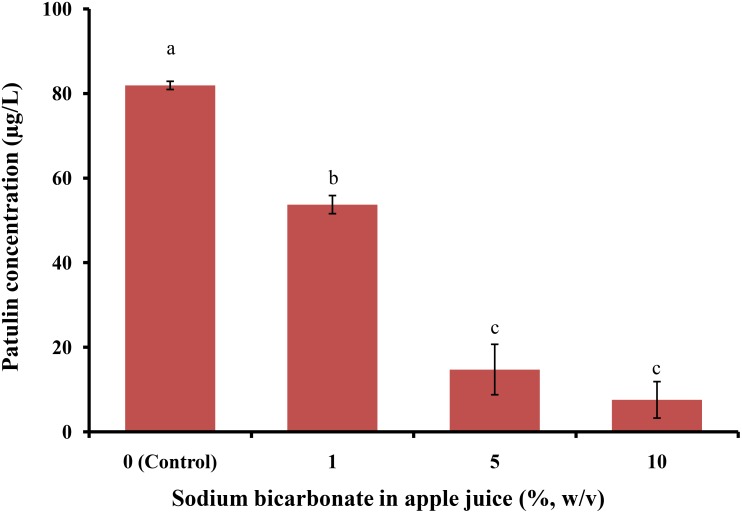
Patulin content in apple juice after treatment with sodium bicarbonate. Bars represent the standard deviation of the mean of three independent experiments. Lowercase letters indicate significant differences at *p* < 0.05.

**FIGURE 5:**
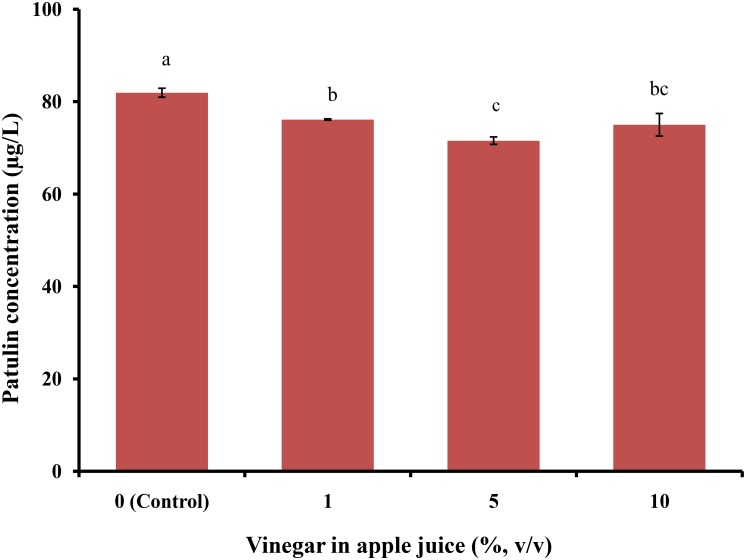
Patulin content in apple juice after treatment with vinegar. Bars represent the standard deviation of the mean of three independent experiments. Lowercase letters indicate significant differences at *p* < 0.05.

Patulin levels in apple juice spiked with PAT (100 μg/L) without sodium bicarbonate were 81.92 ± 0.97 μg/L; however, after treatment with 1, 5, and 10% sodium bicarbonate, they were 53.73 ± 2.14, 14.73 ± 5.96, and 7.55 ± 4.31 μg/L, respectively (**Figure 4**). The rate of PAT reduction increased with an increase in sodium bicarbonate levels in apple juice samples. All sodium bicarbonate-treated apple juice samples were significantly different from the controls (*p* < 0.05). On treating spiked apple juice samples with vinegar (1, 5, and 10%), PAT levels reduced from 81.92 ± 0.97 μg/L (control; **Figure 5**) to 76.13 ± 0.15, 71.54 ± 0.81, and 74.98 ± 2.45 μg/L, respectively.

**FIGURE 6:**
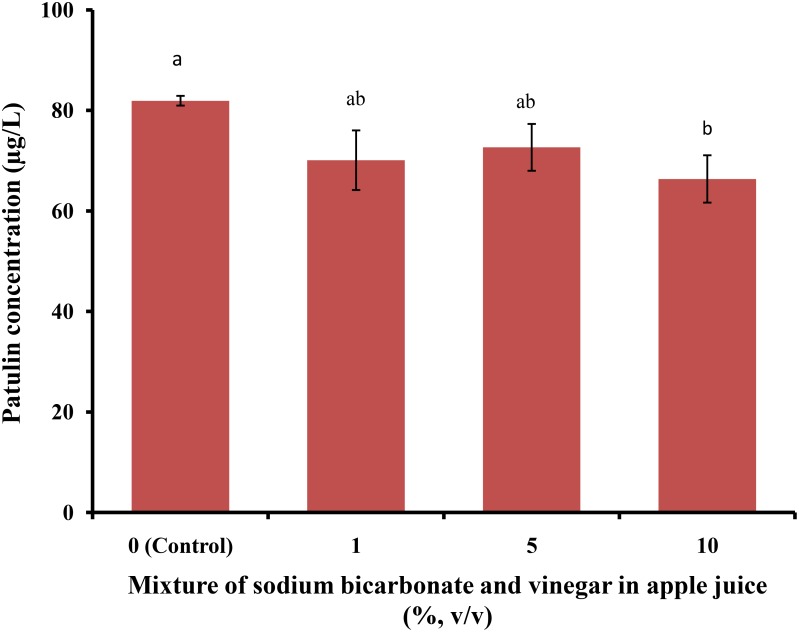
Patulin content in apple juice after treatment with a mixture of sodium bicarbonate and vinegar. Bars represent the standard deviation of the mean of three independent experiments. Lowercase letters indicate significant differences at *p* < 0.05.

Since sodium bicarbonate yielded a significant reduction in PAT levels in spiked apple juice samples as compared to citric acid and vinegar, we analyzed a combination of sodium bicarbonate and vinegar (pre-prepared mixed solution at 1, 5, and 10%) to treat PAT-spiked apple juice samples. As shown in **Figure 6**, after allowing the reaction to proceed at 25°C at 150 rpm for 12 h, PAT levels changed from 81.92 ± 0.97 μg/L (pre-treatment) to 70.08 ± 5.93, 72.64 ± 4.66, and 66.35 ± 4.71 μg/L, respectively. Overall, the combination of sodium bicarbonate and vinegar at 1, 5, and 10% did not significantly reduce PAT; moreover, a 10% mixture also yielded negligible effects. Therefore, we concluded that the tested mixture lacked the anticipated synergistic effect for PAT reduction in apple juice.

**FIGURE 7:**
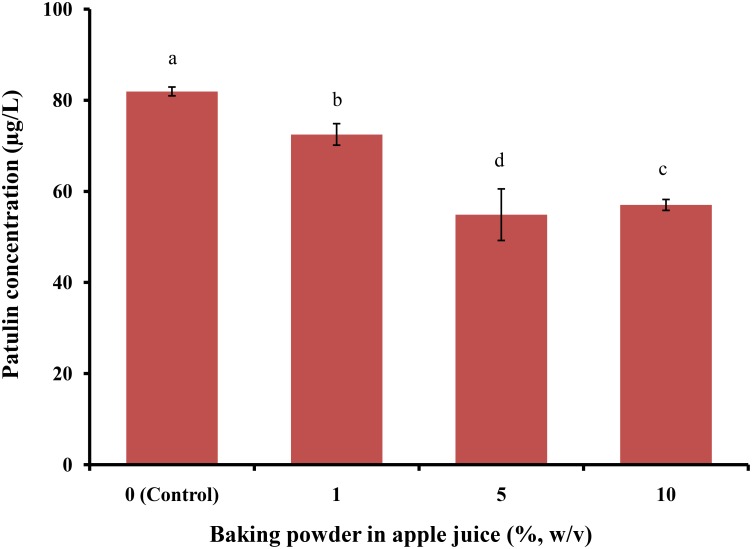
Patulin content in apple juice after treatment with baking powder. Bars represent the standard deviation of the mean of three independent experiments. Lowercase letters indicate significant differences at *p* < 0.05.

Baking powder has recently gained increasing interest in fruit pre-treatments; therefore, we assessed the effect of 1, 5, and 10% of baking powder for reducing PAT content in apple juice samples, which reduced PAT levels to 72.48 ± 2.36, 54.86 ± 5.66, and 57.00 ± 1.2 μg/L, respectively (**Figure 7**). Overall, treatment with baking powder significantly reduced PAT levels, compared to the control (*p* < 0.05).

### Comparative Effects of UV Irradiation on PAT Reduction in Apple Juice

**FIGURE 8:**
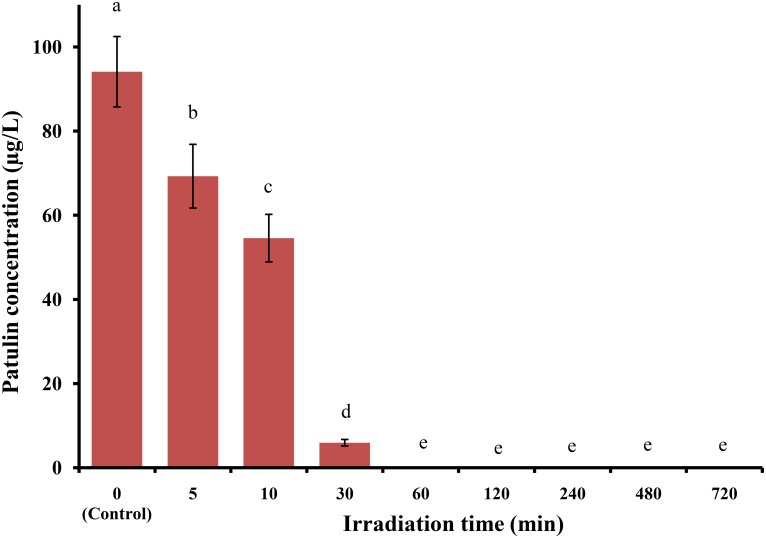
Patulin content in apple juice after UV irradiation. Bars represent the standard deviation of the mean of three independent experiments. Lowercase letters indicate significant differences at *p* < 0.05.

Significant temporal reductions in PAT levels in apple juice (spiked with 100 μg/L PAT) were observed upon UV irradiation. PAT-spiked apple juice samples were UV-irradiated for different time intervals (5, 10, 30, 60, 120, 240, 480, and 720 min), and PAT levels reduced from 94.11 ± 8.37 μg/L (**Figure 8**) to 69.28 ± 7.57, 54.55 ± 5.65, and 5.92 ± 0.78 μg/L after 5, 10, and 30 min, respectively. Interestingly, after 30 min of UV exposure, PAT was not detected in spiked apple juice samples (**Figure 8**). These results confirmed that UV irradiation was the best method. Since this method requires a special UV-irradiation apparatus and energy consumption, a comparison has been made with food-grade additives for reduced PAT levels. The present results indicate that among all tested food-grade additives, sodium bicarbonate yielded significantly higher PAT reduction (7.55 ± 4.31 μg/L), which was comparatively similar to 30 min of UV irradiation (5.92 ± 0.78 μg/L). Therefore, a food-grade additive sodium bicarbonate might be a useful alternative to UV radiation for reducing PAT content in apple juice samples.

### Assay Validation

In this study, validation of the analytical method displayed adequate linearity. The linear regression coefficient of the standard solution curve (*y* = 0.0035*x* + 0.0006) for PAT within the concentration range of 0–700 ng/mL was 0.999. The LOD value obtained was 1.84 ng/mL, and the LOQ was 3.07 ng/mL. The results of the relative standard deviation (RSD%) indicated that the method was compatible, since it yielded high precision with RSD < 20%.

### Changes in Physicochemical Properties of Apple Juice After Sodium Bicarbonate Treatment and UV Irradiation

It is important to assess the physicochemical parameters of treated samples before final validation for using any food-grade additive in processed or unprocessed food products. Since sodium bicarbonate was the most effective additive to reduce PAT content in apple juice samples, various physicochemical parameters, including total soluble solids, pH, turbidity, and color were assessed after apple juice was treated with sodium bicarbonate. As shown in **Table 1**, increasing sodium bicarbonate concentration resulted in an increase in soluble solids, pH, and turbidity of apple juice. Significant differences (*p* < 0.05) were observed with soluble solids, pH, turbidity, *a*^∗^, and *b*^∗^, compared to the control. As shown in the results, although sodium bicarbonate treatment effectively reduced PAT content in spiked apple juice samples, it caused apple juice to taste bitter and deepened the red color of the sample, possibly also affecting its nutritive quality (Korean Food Additives Codex, 2016). Based on these observations, Korean Food Additives Codex (2016) has recommended the addition of citric acid while using sodium bicarbonate as a food additive. Therefore, we hypothesized that citric acid had a possibility of recovering apple juice quality on treatment with sodium bicarbonate.

**Table 1:** Quantification of soluble solids, pH, turbidity, and color change in apple juice on addition of sodium bicarbonate at different concentrations.

Amount (%, w/v)	Soluble solids (°Brix)	pH	Turbidity (λ = 600 nm)	Color		
				*L*^∗^	*a*^∗^	*b*^∗^
0 (Control)	15.00 ± 0.00^c^	3.40 ± 0.10^c^	0.05 ± 0.00^d^	36.50 ± 1.53^ab^	−0.25 ± 0.19^d^	6.08 ± 1.33^b^
1	15.00 ± 0.00^c^	7.43 ± 0.01^b^	0.07 ± 0.00^c^	34.81 ± 1.20^c^	3.13 ± 0.06^c^	10.95 ± 0.16^a^
5	17.20 ± 0.12^b^	7.91 ± 0.06^a^	0.08 ± 0.00^a^	35.69 ± 1.51^bc^	3.57 ± 0.62^b^	10.65 ± 2.21^a^
10	19.07 ± 0.12^a^	7.98 ± 0.00^a^	0.08 ± 0.00^b^	37.25 ± 1.35^a^	3.94 ± 0.15^a^	11.30 ± 1.16^a^

*Data presented are the mean of repeated experiments ± standard deviation, and different lower superscripts in each column indicate significant differences at p < 0.05.*

Furthermore, comparative effects of UV irradiation on the physicochemical parameters of apple juice are enlisted in **Table 2**. The total soluble solids increased steadily with an increasing UV irradiation time. Changes in pH and turbidity were negligible, regardless of UV irradiation time. Regarding color values, values of lightness (*L*^∗^) and redness (*a*^∗^) were similar to those of the control; however, yellowness (*b*^∗^) tended to decrease, compared with that of the control. An increase in UV irradiation time after 30 min resulted in changes in soluble solids and acceptable color values in apple juice. Considering these negative effects of sodium bicarbonate and UV irradiation on the physicochemical properties of apple juice, we considered the suggestion of the Korean Food Additives Codex (2016) regarding the addition of citric acid along with sodium bicarbonate in apple juice samples to achieve the original color and taste after treatment *via* evaluation of the sensory scores with an experienced panel of food scientists.

**Table 2:** Quantification of soluble solids, pH, turbidity, and color change in apple juice after UV irradiation.

Irradiation time (min)	Soluble solids (°Brix)	pH	Turbidity (λ = 600 nm)	Color		
				*L*^∗^	*a*^∗^	*b*^∗^
0 (Control)	14.47 ± 0.06^f^	3.84 ± 0.01^a^	0.04 ± 0.00^a^	37.05 ± 0.01^b^	−0.85 ± 0.15^b^	7.72 ± 0.38^a^
5	14.47 ± 0.06^f^	3.84 ± 0.01^ab^	0.04 ± 0.00^a^	37.62 ± 1.39^b^	−0.64 ± 0.21^a^	3.96 ± 0.50^b^
10	14.50 ± 0.00^f^	3.84 ± 0.01^ab^	0.04 ± 0.00^a^	37.86 ± 1.33^b^	−0.71 ± 0.06^ab^	3.77 ± 0.07^bc^
30	14.53 ± 0.06^f^	3.84 ± 0.01^a^	0.04 ± 0.00^a^	37.24 ± 0.54^b^	−0.80 ± 0.07^ab^	3.45 ± 0.10^c^
60	14.70 ± 0.00^e^	3.85 ± 0.00^a^	0.04 ± 0.01^a^	37.64 ± 1.62^a^	−0.88 ± 0.09^b^	3.47 ± 0.20^c^
120	15.23 ± 0.06^d^	3.83 ± 0.01^ab^	0.05 ± 0.01^a^	38.41 ± 0.41^a^	−0.98 ± 0.75^b^	2.36 ± 0.19^e^
240	16.03 ± 0.06^c^	3.83 ± 0.01^ab^	0.05 ± 0.01^a^	38.34 ± 0.42^a^	−0.75 ± 0.13^ab^	2.88 ± 0.07^d^
480	18.47 ± 0.06^b^	3.80 ± 0.02^c^	0.05 ± 0.01^a^	38.03 ± 0.48^a^	−0.86 ± 0.01^b^	2.46 ± 0.04^de^
720	21.30 ± 0.00^a^	3.82 ± 0.01^b^	0.04 ± 0.00^a^	38.42 ± 0.10^a^	−0.81 ± 0.03^ab^	2.51 ± 0.14^de^

*Data presented are the mean of repeated experiments ± standard deviation, and different lower superscripts in each column indicate significant differences at p < 0.05.*

### Sensory Evaluation of Apple Juice on Addition of Citric Acid After Treatment With Sodium Bicarbonate

Although sodium bicarbonate significantly reduced PAT levels in apple juice, changes in physicochemical parameters were observed in soluble solids, pH, turbidity, and color. Therefore, sensory evaluation was warranted to compare physicochemical and sensory properties of apple juice treated with sodium bicarbonate and citric acid with those of untreated apple juice. The test was conducted by a panel of 15 individuals (20–35 years old). They were provided two types of apple juice: (1) untreated (control) and (2) samples treated with 5% sodium bicarbonate and citric acid. Most of the panelists (80%) reported that the two samples had the same color and odor but tasted different; moreover, they preferred untreated samples to treated samples, overall.

Regarding descriptive sensory analysis, a few panelists reported that apple juice treated with sodium bicarbonate and citric acid was salty, while untreated apple juice was sweet. However, a few panelists reported that the taste of apple juice treated with sodium bicarbonate and citric acid also fulfilled the criteria for acceptability. Therefore, sodium bicarbonate can be considered an apple juice additive for PAT reduction.

## Discussion

Heat-resistant mycotoxin PAT has been an interesting concern in fresh apples, apple cider, and apple juice products owing to serious long-term health complications reported in laboratory animals (Assatarakul et al., 2012). Through selective elimination of contaminated apples, PAT can also be eliminated or reduced during apple harvesting, processing, and storage (Moake et al., 2005). Based on good agricultural practices (Codex Alimentarius Commission, 2003), the Joint FAO/WHO Food Standards Program recommends that careful apple selection before processing is one of most effective means to control PAT content. Indeed, a selective trimming step can reduce PAT burdens up to 99% (Lovett et al., 1975). Chemical degradation of PAT is another potential solution, since the method is easily accommodated into traditional processing streams. However, the use of chemical agents solely for mycotoxin reduction is currently not permitted, although many chemicals can also serve other purposes; for example, ascorbic acid (Vitamin C) is commonly used to prevent oxidation or browning (Root and Barrett, 2005).

Furthermore, application of biological control methods (lactic acid bacteria or enzymatic degradation based) can significantly reduce PAT levels in apple-based products with no significant effects on juice quality characteristics including °Brix, acidity, color, and clarity (Yue et al., 2011). Further studies are required to completely understand the processes involved and to determine how to safely incorporate these methods into food processing. UV radiation is an approved non-thermal method for the preservation of fruit juices in both Canada and the United States (FDA). In the present study, we assessed all food-grade agents (sodium bicarbonate, vinegar, a mixture of sodium bicarbonate and vinegar, citric acid, baking powder, and UV treatment) for significant and comparative reductions of PAT content in apple juice samples.

Humer et al. (2016) reported the potential effect of 5% citric acid solution to reduce the levels of deoxynivalenol in contaminated feed samples. Deoxynivalenol levels were reduced to 46% of the initial level after 48 h of soaking with 5% citric acid solution (Humer et al., 2016). However, in the present study, neither 5 nor 10% of citric acid yielded significant PAT reductions after 12 h, probably owing to the varied chemical composition in food matrixes. Sholberg et al. (2000) reported the potential effects of vinegar vapor fumigation on the reduction of the size of apple surface lesions, contaminated with *Penicillium expansum* (10^5^ CFU/mL). Furthermore, complete reduction of mycelia growth of the aflatoxin producing *Aspergillus flavus* after 90 min of exposure to vinegar vapor was reported (Pornpukdeewattana et al., 2017). However, in the present study, PAT content did not decrease to below 70 μg/L in the vinegar-treated apple juice samples. Therefore, vinegar could not be considered an appropriate additive for PAT reduction in apple juice.

Moreover, sodium bicarbonate can reduce germinating *P. expansum*, which is known to produce PAT, by up to 80% (Lai et al., 2015). Furthermore, Lai et al. (2015) and Puel et al. (2007) reported that sodium bicarbonate downregulated of methyl salicylic acid synthase and isoepoxydon dehydrogenase genes in *P. expansum*. Similarly, in the present study, sodium bicarbonate displayed its potential effects on PAT reduction in apple juice samples. According to the Ministry of Food and Drug Safety (2010), PAT has characteristics to be hydrolyzed in alkaline medium. Therefore, we assumed that PAT could be reduced owing to the alkaline nature of sodium bicarbonate. Furthermore, as sodium bicarbonate primarily constitutes baking powder, Palou et al. (2001) reported that sodium bicarbonate solution inhibits the growth of *Penicillium italicum* by more than 50%. Hence, we assumed that baking powder may also be effective ingredient for PAT reduction in apple juice. However, consequently, PAT reduction on treating apple juice with baking powder was lower than that with sodium bicarbonate.

Finally, all additive-based treatments for PAT reduction were compared with UV irradiation treatment, which also significantly reduced PAT levels. Similarly, Ibarz et al. (2014) reported that the irradiation distance from the lamp to the surface of the PAT-spiked samples was 22.5 cm, and 60 min and 120 min of irradiation could reduce 50% and 70% of the initial content of PAT, respectively. However, in the present study, the irradiation distance was 11 cm, and PAT was completely reduced after 60 min of UV irradiation; this enhancing effect may have resulted from the distance between the food sample and the UV lamp, which could affect PAT reduction (Tikekar et al., 2014).

On confirming that sodium bicarbonate as a food-grade additive is potentially effective in reducing PAT content in apple juice samples, the color and taste of apple juice should not be affected. Therefore, we also quantified the sensory and color properties of the apple juice samples after treatment. Zhu et al. (2014) reported that soluble solids, pH, *L*^∗^, *a*^∗^, and *b*^∗^ values of apple juice after 90 min UV irradiation were similar to those of the control. However, ascorbic acid levels in apple juice after UV irradiation reduced to 45% of the control (Zhu et al., 2014). Similarly, in the present study, soluble solids, pH, turbidity, *L*^∗^, and *a*^∗^ were similar to those of the control within 30 min of irradiation. However, soluble solids increased after 120 min of irradiation. These observations confirmed that UV irradiation affects the quality of apple juice, thereby generating interest in investigating methods to recover apple juice quality after UV irradiation.

In accordance with these findings, we treated apple juice with sodium bicarbonate, followed by citric acid to recover the sensory, color, and nutritive properties of apple juice samples (Korean Food Additives Codex, 2016). Finally, these tests confirmed that apple juice treated with sodium bicarbonate followed by addition of citric acid was efficient in reducing PAT contamination in apple juice without any adverse effects on color and sensory acceptability.

## Conclusion

This study aimed to determine an optimal method for PAT reduction in apple juice. The experiments involved measurement of PAT levels in apple juice after treatment with citric acid, sodium bicarbonate, vinegar, a mixture of sodium bicarbonate and vinegar, baking powder, and UV irradiation. Among these treatments, treatment with 5% and 10% sodium bicarbonate decreased PAT content below the maximum recommended concentration (50 μg/kg) in apple juice. UV irradiation for more than 30 min also decreased PAT content below 50 μg/kg. Thus, sodium bicarbonate and UV irradiation were the most effective treatments; however, UV irradiation affected the quality of apple juice, i.e., the yellowness (*b*^∗^) value of apple juice decreased after UV irradiation, compared with that of the control. Sodium bicarbonate has been used as an acidity regulator and is safe for consumption. Sodium bicarbonate treatment affected soluble solids, pH, redness (*a*^∗^), and yellowness (*b*^∗^) of apple juice. However, the Korean Food Additives Codex recommends the addition of citric acid along with sodium bicarbonate in food. Therefore, sensory evaluation was conducted to compare physicochemical and sensory properties of apple juice treated with sodium bicarbonate and citric acid with those of untreated apple juice. Upon a panel-based evaluation of sensory characteristics, 80% of panelists reported that two samples of apple juice were same in color and odor, but tasted different. Additionally, panelists preferred untreated apple juice, overall; however, a few panelists reported that the taste of apple juice treated with sodium bicarbonate and citric acid was acceptable. Therefore, sodium bicarbonate can be considered an effective additive in apple juice for PAT reduction.

## Author Contributions

MKK and YO performed the experiments. SS, YO, SHC, and MHK designed and interpreted the data. MKK, SS, and MHK drafted the manuscript. All authors approved the final version of the manuscript.

## Conflict of Interest Statement

The authors declare that the research was conducted in the absence of any commercial or financial relationships that could be construed as a potential conflict of interest.
